# A Two-Tube Multiplex Reverse Transcription PCR Assay for Simultaneous Detection of Viral and Bacterial Pathogens of Infectious Diarrhea

**DOI:** 10.1155/2014/648520

**Published:** 2014-03-10

**Authors:** Ji Wang, Ziqian Xu, Peihua Niu, Chen Zhang, Jingyun Zhang, Li Guan, Biao Kan, Zhaojun Duan, Xuejun Ma

**Affiliations:** ^1^Key Laboratory for Medical Virology, Ministry of Health, National Institute for Viral Disease Control and Prevention, Chinese Center for Disease Control and Prevention, No. 155, Changbai Road, Changping District, Beijing 102206, China; ^2^National Institute for Infectious Disease Control and Prevention, Chinese Center for Disease Control and Prevention, Beijing 102206, China

## Abstract

Diarrhea caused by viral and bacterial infections is a major health problem in developing countries. The purpose of this study is to develop a two-tube multiplex PCR assay using automatic electrophoresis for simultaneous detection of 13 diarrhea-causative viruses or bacteria, with an intended application in provincial Centers for Diseases Control and Prevention, China. The assay was designed to detect rotavirus A, norovirus genogroups GI and GII, human astrovirus, enteric adenoviruses, and human bocavirus (tube 1), and *Salmonella*, *Vibrio parahaemolyticus*, diarrheagenic *Escherichia coli*, *Campylobacter jejuni*, *Shigella*, *Yersinia,* and *Vibrio cholera* (tube 2). The analytical specificity was examined with positive controls for each pathogen. The analytical sensitivity was evaluated by performing the assay on serial tenfold dilutions of in vitro transcribed RNA, recombinant plasmids, or bacterial culture. A total of 122 stool samples were tested by this two-tube assay and the results were compared with those obtained from reference methods. The two-tube assay achieved a sensitivity of 20–200 copies for a single virus and 10^2^-10^3^ CFU/mL for bacteria. The clinical performance demonstrated that the two-tube assay had comparable sensitivity and specificity to those of reference methods. In conclusion, the two-tube assay is a rapid, cost-effective, sensitive, specific, and high throughput method for the simultaneous detection of enteric bacteria and virus.

## 1. Introduction

Diarrhea caused by viral and bacterial infections is a major health problem in developing countries and the clinical presentation of the patients with diarrhea symptoms is not generally indicative of a specific virus or bacteria [1, 2]. In China, the analysis of reported infectious diarrhea conducted by China Centre for Disease Prevention and Control (CCDC) in 2011 showed that the main pathogens of bacterial diarrhea were* Salmonella*,* Vibrio parahaemolyticus*, diarrheagenic* Escherichia coli*, and* Campylobacter jejuni*, accounting for 48.43% (1570/3242), 32.20% (1044/3242), 8.57% (278/3242), and 2.41% (78/3242), respectively. Rotavirus cases had the highest proportion of viral cases with 97.35% (55185/56687), followed by enteric adenovirus, norovirus, and human astrovirus, accounting for 2.60% (1476/56687), 0.04% (25/56687), and 0.01% (1/56687), respectively. In total, these pathogens caused 99.55% cases of infectious diarrhea in China in 2011 [3].* Shigella* and* Vibrio cholera *were not counted in this report, but they can also cause diarrhea [4]. The existing studies indicated that human bocavirus and* Yersinia *were also associated with acute gastroenteritis [5, 6].

Conventional diagnostic methods for routine detection of enteric pathogens within the clinical microbiology setting rely on microscopy, culture, and enzyme immunoassays [7]. However, these procedures are either laborious or have limited sensitivity and specificity. Molecular methodologies based on polymerase chain reaction (PCR) and reverse transcription (RT-PCR) provide powerful tools [8], which have markedly improved the detection of enteric pathogens [9]. In the last few years, the multiplex real-time polymerase chain reaction (RT-PCR) has been successfully performed to identify different enteric pathogens simultaneously [1, 10–12]. However, these methods are either low throughput (4 pathogens/tube at most) or expensive (fluorescence probe), which does not allow rapid screening of large numbers of stool samples. Furthermore, almost all of the reported multiplex PCR methods are able to detect either viral pathogens [13–15] or bacterial pathogens [16], but few studies reported the simultaneous detection of viral and bacterial pathogens within the same sample, which is essential to elucidate the potential synergy between enteric virus and bacteria and reveal the clinical relevance between viral and bacterial infections. Although several multiplex molecular assays such as Luminex GPP and Seegene Diarrhea ACE are commercially available, the extremely high price made them unaffordable methods for routine use in provincial Centers for Diseases Control and Prevention, China. Therefore, a rapid, sensitive, specific, and cost-effective diagnostic method which detects virus and bacteria simultaneously would be highly preferred for routine laboratory testing.

Here, we describe the development of a one-step multiplex PCR assays (two-tube assay) for the simultaneous detection of 13 most commonly found diarrhea-causative viruses or bacteria. Tube 1 is used for the detection of 6 diarrhea-associated viruses including rotavirus A (RVA), noroviruses (NoVs) genogroups GI and GII, human astrovirus (HAstV), enteric adenoviruses (EAds), and human bocavirus (HBoV) (Qiaxcel-V assay), and tube 2 is used for the detection of 7 diarrhea-associated bacteria including* Salmonella*,* Vibrio parahaemolyticus*, diarrheagenic* Escherichia coli*,* Campylobacter jejuni*,* Shigella*,* Yersinia*, and* Vibrio cholera* (Qiaxcel-B assay) using the same samples from patients with gastroenteritis. The analytical specificity and sensitivity were examined and the clinical performance of the two-tube assay was evaluated by comparing the results with those obtained from reported PCR assay or commercial kits.

## 2. Materials and Methods

### 2.1. Virus, Strains, and Clinical Samples

Two test panels of viral and bacterial samples used in this study are listed in Tables 1 and 2. The virus test panel consisted of 47 clinical samples previously determined by PCR and sequencing conducted by the Diarrhea Department, National Institute for Viral Disease Control and Prevention (DD-IVDC). The bacterial test panel consisted of 27 preserved strains or clinical isolates previously determined by cultural and molecular identification by the Diarrhea Department, National Institute for Infectious Disease Control and Prevention (DD-ICDC). A total of additional 122 fecal samples were selected from the collection obtained during routine virus and bacteria surveillance conducted by DD-IVDC. All of the fecal specimens and clinical data were collected from January 2012 to December 2012 from hospitalized children under 5 years old diagnosed of acute diarrhea. All aspects of the study were performed in accordance with national ethics regulations and approved by the Institutional Review Boards of CCDC. Children's parents were apprised of the study's purpose and of their right to keep information confidential. Written consent was obtained from children's parents. RVA was identified using a commercial ProSpecT rotavirus ELISA kit (Oxoid, Hants, UK). Norovirus and enteric adenovirus were identified using reported multiplex PCR assay while the HAstV and HBoV were identified using monoplex PCR followed by sequencing [13, 17–19] at DD-IVDC. All specimens were processed by routine isolation/culture to identify different enteropathogenic bacteria at DD-ICDC. The diarrheagenic* Escherichia coli* were identified using multiplex real-time PCR assay [20]. In order to facilitate the presentation, these methods are defined as “reference methods” in the following text, and the test results of reference methods are called “reference results.”

### 2.2. Primers

Primers for* Vibrio cholera*, Eads, and HBoV were designed from conserved regions of the viral and bacterial genomes using Primer-Premier software version 5.0. For all primer sequences, BLAST analysis was performed to ensure specificity and no sequence cross-reactivity was observed. Primers for other targeted virus and bacteria were adapted or modified from previous references. In total, 14 pairs of chimeric primers sequences, the target genes, and the amplicon sizes are listed in Table 3. One pair of universal primers was adapted from our previous studies [21]. The chimeric primers consisted of a gene-specific sequence fused at the 5′ end to the universal sequence; thus, all of the chimeric primers are with similar annealing temperatures to assure the approximate amplification efficiency.

### 2.3. Nucleic Acid Extraction

Total nucleic acid was extracted from 200 *μ*L of a 10% fecal suspension prepared in normal saline using the MasterPure Complete DNA and RNA purification kit (Epicenter Technologies, Madison, WI) according to the manufacturer's instructions. The extracts were eluted in 50 *μ*L of DNase- and RNase-free water and stored at −80°C.

### 2.4. Multiplex PCR

Two multiplex PCR assays were developed in this study. Six enteric viruses were detected by Qiaxcel-V assay in tube 1 while the 7 enteric bacteria were detected by Qiaxcel-B assay in tube 2 simultaneously.

For the Qiaxcel-V assay, the multiplex RT-PCR was performed with a One-Step RT-PCR kit (Qiagen, Hilden, Germany) in a 25 *μ*L volume, containing 1.25 pmol each of the forward and reverse viral chimeric primer mix, 12.5 pmol each of the forward and reverse universal primer mix, 4 *μ*L of template nucleic acid and 0.1 *μ*L of ribonuclease inhibitor (Takara, Dalian, China), and RNase-free water. The RT-PCR mixture was subjected to the following amplification conditions: 50°C for 30 min and 95°C for 15 min, followed by 10 cycles of 95°C for 30 s, 56°C for 30 s, and 72°C for 60 s; 10 cycles of 95°C for 30 s, 68°C for 30 s, and 72°C for 60 s; 20 cycles of 95°C for 30 s, 50°C for 30 s, and 72°C for 60 s and a final incubation of 72°C for 3 min. For the Qiaxcel-B assay, the multiplex PCR was performed with a Multiplex PCR kit (Qiagen, Hilden, Germany) in a 25 *μ*L volume, containing 1.25 pmol each of the forward and reverse bacterial chimeric primer mix, 12.5 pmol each of the forward and reverse universal primer mix, and 3 *μ*L of template nucleic acid. The multiplex PCR mixture was subjected to the following amplification conditions: 95°C for 15 min, followed by 10 cycles of 95°C for 30 s, 65°C for 90 s, and 72°C for 60 s; 25 cycles of 95°C for 30 s, 70°C for 90 s, and 72°C for 60 s and a final incubation of 72°C for 3 min. The thermal cycling was performed using PCR Amplifier (Thermo Electron Corp., Vantaa, Finland) followed by the detection of amplified DNA products by agarose gel (3.0%) or capillary electrophoresis using QIAXCEL and DNA Screening kit (Qiagen, Hilden, Germany).

### 2.5. Analytical Specificity and Sensitivity

The analytical specificity of the two-tube assay was determined by the testing of two test panels individually. DNA, RNA, or bacterial culture with a known concentration was prepared to evaluate the analytical sensitivity of the two-tube assay.

For the Qiaxcel-V assay, the analytical sensitivity for each virus was examined using serial tenfold dilutions ranging from 2 × 10^1^ to 2 × 10^5^ copies of recombinant plasmids for DNA virus and in vitro transcribed RNA for all RNA viruses. The target genes of 6 viruses were amplified with specific primers using the virus test panel (Table 1). Then the products were purified and ligated to pGEM-T vector (Promega, Madison, WI) to construct recombinant plasmids. For RNA viruses, the plasmids amplified by* E. coli *were linearized with Spe I and in vitro transcribed from the T7 promoter using Ribo-MAX large scale RNA Production System T7 (Promega, Madison, WI). The RNA copy number was calculated after measuring the purified RNA concentration by spectrophotometry using Eppendorf Biophotometer (Eppendorf AG, Hamburg, Germany). For DNA viruses, the copy number of plasmids was calculated after measuring the plasmid concentration using Eppendorf Biophotometer (Eppendorf AG, Hamburg, Germany). Tenfold serial dilutions of these RNA/DNA templates with known copy numbers (2 × 10^1^ to 2 × 10^5^ copies/mL) were used to evaluate the analytical sensitivity of the Qiaxcel-V assay.

For the Qiaxcel-B assay, each reference strain of bacterial test panel (Table 2), except for* Vibrio cholera*, was serially diluted 10-fold with 0.9% NaCl in the logarithmic phase of growth. The bacterial dilutions were plated onto agar plates and incubated. Thereafter, the colony-forming units (CFU) were counted in duplicate and the DNA in the bacterial dilutions was extracted with MasterPure Complete DNA and RNA purification kit (Epicenter Technologies, Madison, WI) according to the manufacturer's instructions. The DNA templates were used to evaluate the analytical sensitivity of the Qiaxcel-B assay. The analytical sensitivity for* Vibrio cholera *was tested in the same way of DNA virus (quantitative recombinant plasmids) due to the unavailability of live* Vibrio cholera*.

The analytical sensitivity of Qiaxcel-V assay and Qiaxcel-B assay was examined, respectively, by using quantitative premixed templates. To confirm the reproducibility of this method, intra-assay (each sample tested three times within an experiment) and interassay (each sample tested one time in three different experiments) precision were evaluated.

### 2.6. Diagnostic Specificity and Sensitivity

A total of 122 samples were tested by two-tube assay (Qiaxcel-V assay and Qiaxcel-B assay). The diagnostic specificity and sensitivity were determined in comparison to the reference results (Table 4). The discordant results between the two-tube assay and the reference method were resolved by a third monoplex PCR and sequencing [14, 22].

### 2.7. Statistical Analysis

All statistical analyses were performed using Statistical Package for Social Sciences (SPSS) software (version 13.0) for Windows. The *χ* ^2^-test and McNemar's test were conducted to measure the sensitivity, specificity, and the detection agreement of two-tube assay with the reference results.

## 3. Results

### 3.1. Analytical Specificity and Sensitivity

**Figure 1:**
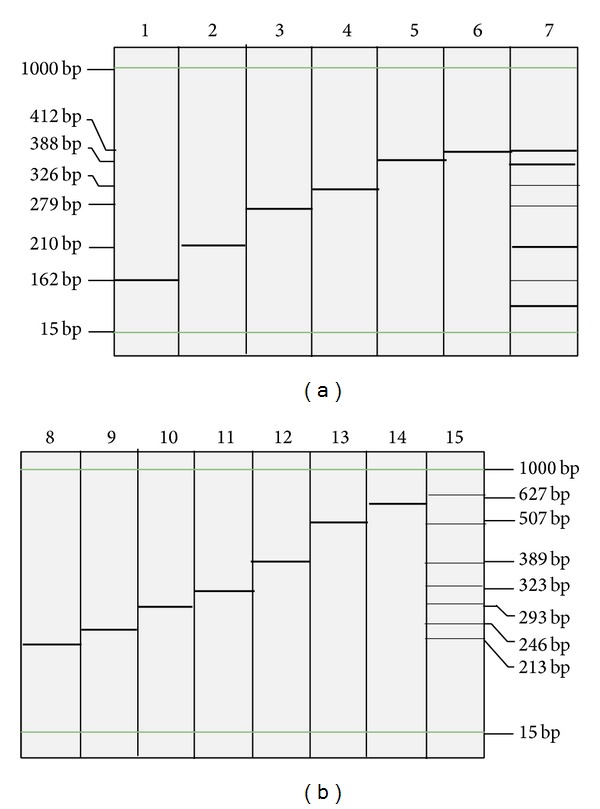
Electrophoresis results of the PCR products on automatic electrophoresis. All of the targets were identified successfully, and no mispriming was observed in either tube. Lanes 1 to 6 in part (a): NoV GI (162 bp), RVA (210 bp), NoV GII (279 bp), HAstV (326 bp), EAds (388 bp), and HBoV (412 bp), respectively. Lane 7: PCR products of six premixed viral targets in tube 1 (Qiaxcel-V assay). Lanes 8 to 14 in part (b):* Shigella* (213 bp),* Vibrio parahaemolyticus* (246 bp), diarrheagenic* Escherichia coli* (293 bp),* Salmonella* (323 bp),* Yersinia* (389 bp),* Vibrio cholera* (507 bp), and* Campylobacter jejuni* (627 bp). Lane 15: PCR products of seven premixed bacterial targets in tube 2 (Qiaxcel-B assay).

**Figure 2:**
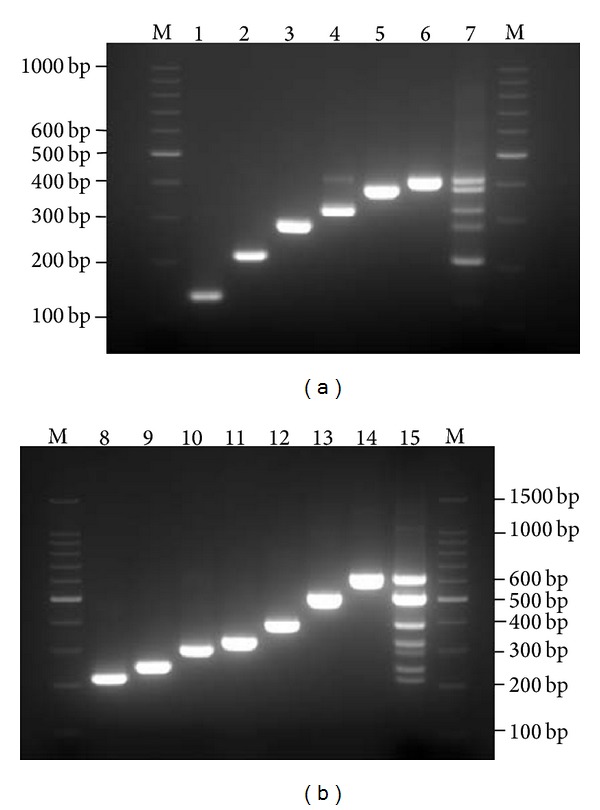
Electrophoresis results of the PCR products on 3% agarose gel. Agarose gel electrophoresis demonstrated the expected PCR product sizes of 6 viruses and 7 bacteria by the novel two-tube multiplex PCR method. The results shown in lanes 1 to 14 were in the same order as in Figure 1. M: standard 100 bp DNA ladder marker.

The analytical specificity result was shown in Tables 1 and 2; the result of inclusivity and exclusivity experiments demonstrated no cross-reactivity among the target organisms, closely related organisms, or commonly encountered organisms. The amplified DNA products were detected both on capillary electrophoresis Qiaxcel (Figure 1) and 3.0% agarose gel (Figure 2). The results revealed that the expected size of each pathogen-specific amplicon was observed and separated clearly from the other targets. No mispriming (primer dimer) or other amplification was observed in either tube.

The analytical sensitivity for norovirus GII, RVA, and enteric adenovirus was 20 copies/reaction and 200 copies/reaction for the other 3 viruses. The sensitivity of the six premixed viral templates was 2000 copies/reaction (for simultaneous detection of six targets) in the Qiaxcel-V assay (data not shown). For the Qiaxcel-B assay, the individual sensitivity for bacterial dilutions was 4.26 × 10^3^ CFU/mL for* Salmonella*, 3.22 × 10^2^ CFU/mL for* Vibrio parahaemolyticus*, 5.17 × 10^3^ CFU/mL for* Escherichia coli*, 4.31 × 10^3^ CFU/mL for* Campylobacter jejuni*, 2.88 × 10^3^ CFU/mL for* Shigella*, 5.64 × 10^2^ CFU/mL for* Yersinia,* and 20 copies/reaction for* Vibrio cholera*. The analytical sensitivity of simultaneous detection of the seven premixed bacterial templates was about one order of magnitude higher than that of individual target. The coefficient of variation (CV) for interassay and intra-assay ranged from 3.25% to 7.98% (data not shown).

### 3.2. Diagnostic Specificity and Sensitivity

The clinical performance of the two-tube assay was evaluated using a panel of 122 archived clinical specimens. In comparing the results of the novel two-tube assay with the reference results, no significant difference was found between the detection rates in the clinical evaluation (Table 4). Five norovirus GII-positive samples, one norovirus GI-positive sample, and one* Shigella*-positive sample by reference method were negative by the two-tube assay. Five rotavirus-negative samples and two enteric adenovirus-negative samples identified by reference method were positive by the two-tube assay. In addition, 21 samples identified as coinfection were detected (Table 5). Of the 122 clinical samples tested, the sensitivities of detection of the different pathogens were 100% (RVA), 83.33% (NoV GI), 88.98% (NoV GII), 100% (HAstV), 100% (EAds), 100% (HBoV), 80% (*Shigella*), 100% (*Salmonella*), and 100% (*Escherichia coli*), and the specificities were 94.57%, 100%, 100%, 100%, 98.17%, 100%, 100%, 100%, and 100%, respectively. The positive samples detected only by two-tube assay (5 for RVA and 2 for EAds) were retested and confirmed by sequencing as true positives using other reported primers [14, 22]. Compared with the confirmed result, the specificities of RVA and EAds were both 100%. The agreement was >90% for all of the identified pathogens (except for* Vibrio parahaemolyticus*,* Campylobacter jejuni*,* Yersinia*, and* Vibrio cholera*, where no positive case was found), and the kappa correlation between the two methods was >0.75.

## 4. Discussion

In our previous study, a multiplexed Luminex-based assay [23] to detect seven enteric viruses associated with acute gastroenteritis was developed. However, this method is not likely to be widely adopted in common laboratories due to the limited availability of Luminex equipment. In this study, a novel two-tube assay using automatic electrophoresis was developed and evaluated for the simultaneous detection of 6 viruses and 7 bacteria, with an intended application in provincial Centers for Diseases Control and Prevention.

A multiplex PCR based on the chimeric primer and temperature switch PCR (TSP) strategy has been developed in our laboratory and shown to be effective for the detection of pandemic influenza A H1N1 virus [24], nine serotypes of enteroviruses associated with hand, foot, and mouth disease [25], and sixteen different respiratory virus types/subtypes in a single tube [26]. This strategy was also applied in this study. In addition, two sets of primers (RVA and norovirus GII) were modified from our previous study [23] to achieve better analytical sensitivity. The analytical result indicated that this assay's ability to detect a low concentration of RVA and norovirus GII in biological samples was improved compared to our previous study [23]. The specificity and the sensitivity of the two-tube assay for each viral and bacterial pathogen were comparable to those of previous reports [23, 27, 28].

Viral and bacterial targets were amplified in the same tube in our preliminary experiments, but the bacterial PCR significantly inhibited the amplification of viral targets likely because of the competition of reagent (data not shown). Therefore, the novel assay was composed by two tubes (Qiaxcel-V and Qiaxcel-B) to minimize the decrease in the analytical sensitivity. To optimize the PCR condition, several commercial PCR kits or one-step RT-PCR kit from different companies (Promega, Qiagen, Takara and Invitrogen) was tested in our preliminary experiments. The multiplex PCR kit and the one-step RT-PCR kit from Qiagen revealed the best amplification efficiency under our current protocol.

Of 122 clinical samples tested, positive samples accounted for 77.87% (95/122) of all the samples, including 8.42% (8/95) bacterial-positive samples and 95.80% (91/95) virus-positive samples.

Compared with bacteria, virus causes more gastroenteritis cases, which is consistent with the previous studies [1, 3, 15]. RVA is the principal pathogen causing infectious diarrhea [3], but, in our study, norovirus GII took the highest proportion of viral positive samples, with a detection rate of 43.96% (40/91), followed by RVA with 32.97% (30/91). The reason for this is that among all the norovirus GII-positive samples some of them were collected from local outbreaks, while all of the RVA samples were collected from the sporadic cases of gastroenteritis. The bacteria detected from clinical specimens were* Shigella*,* Salmonella*, and* Escherichia coli*, accounting for 44.44% (4/9), 33.33% (3/9), and 11.11% (1/9), respectively. It should be noted that no sample tested positive for* Vibrio parahaemolyticus*,* Campylobacter jejuni*,* Yersinia*, and* Vibrio cholera* with limited specimens. No pathogens were detected in 27 (22.13%) diarrheal stool samples in this study. This could be due to the infection of other causative pathogens not included in this diagnostic panel, such as parasite, sapovirus,* Staphylococcus aureus*, and other serotypes of* E. coli*.

As shown in Table 5, twenty-one coinfections were observed. Among all pathogens, RVA is the most frequently identified in coinfection with other pathogens (12/21). In addition, all of viral-bacterial coinfections are identified involving RVA, and the ages of these children are older than 12 months. Two reasons may lead to this phenomenon. First, RVA infection is thought to be the reason of enterocyte destruction from the top of intestinal villus [29], which properly increases the risk of opportunistic pathogen infection. Second, the children older than 12 months have the ability of walking and prefer to grab or bite things. This behavior increases the probability of touching the food, toys, and baby stuff which are contaminated by bacteria such as* Salmonella*.

Due to the limited specimens available at this time, only preliminary findings were reported in this study. The associations between mixed infections and severity of diarrhea and the information about the seasonal prevalence of a wide range of viral and bacterial pathogens are not able to be addressed. In addition, clinical data provide no information about the use of antibiotics, which may influence the detection result of bacterial infections. Future study is therefore needed to include larger sample size to further evaluate the clinical performance of this two-tube assay.

The two-tube assay has acceptable turnaround time (TAT) for high throughput analysis of 96 samples. The whole PCR process in two tubes is completed within 2.5 hours, followed by capillary electrophoresis separation (10 min/12 wells). The average turnaround time for processing 96 samples targeting 13 pathogens in one run is less than 5 hours (apart from the nucleic acid extraction step). Additionally, the PCR product of the two-tube assay could be identified and separated in agarose gel (3.0%) clearly; thus, the minimal requirement for the assay's implementation only includes refrigerator, PCR amplifier, agarose gel electrophoresis system, and UV detector, for which the two-tube assay is likely to be widely adopted in the laboratories that equip no capillary electrophoresis such as Qiaxcel.

Though the test panel of the commercial kits (16 pathogens for Luminex xTAG GPP and 15 pathogens for Seegene Diarrhea ACE) is more comprehensive than two-tube assay (13 pathogens), the pathogen compositions of two-tube assay are reasonable enough as the pathogen panel was selected according to the report of routine surveillance program on infectious diarrhea China CDC [3]. Compared to the existing commercial Seeplex Diarrhea ACE Detection kit (Seegene, Seoul, Korea), the reagent cost (the PCR kit and the consumables of Qiaxcel capillary electrophoresis) of the two-tube assay is approximately $8/test versus $120/test in three tubes using Seeplex kit. It is not fair though to compare the material cost to market price; it is a fact that the commercial kit is unaffordable for the use in the routine surveillance of enteric pathogens. Therefore, the two-tube assay demonstrates the advantage of practicability of the potentially wide application.

In conclusion, we present a rapid and high throughput multiplex PCR with high specificity and sensitivity. More importantly, this assay is affordable and convenient for the routine use in primary testing laboratories. As the two-tube assay uses the QIAxcel automated electrophoresis system, which is accessible in most of provincial Centers for Disease Control and Prevention in China, the proposed assay is demonstrated to have great potential for routine surveillance of enteric viral and bacterial infection in China and to improve the capacity for emergency management.

## Figures and Tables

**Table 1:** Virus test panel for the evaluation of specificity of PCR primers.

Isolates	Number of the isolates^b^	Targeted gene loci of virus					
		VP6	RDRP^a^	RDRP	ORF1a	Hexon	VP1
Rotavirus A	9	+	−	−	−	−	−
Noroviruses G1	1	−	+	−	−	−	−
Noroviruses G2	6	−	−	+	−	−	−
Astrovirus	2	−	−	−	+	−	−
Adenovirus	10	−	−	−	−	+	−
Human bocavirus	6	−	−	−	−	−	+
Coxsackie virus 16	2	−	−	−	−	−	−
Enterovirus 71	4	−	−	−	−	−	−
Influenza virus B	3	−	−	−	−	−	−
Influenza virus A	4	−	−	−	−	−	−

^a^RDRP, RNA-dependent RNA polymerase; ^b^all the isolates in this panel were clinical samples.

**Table 2:** Bacterial test panel for the evaluation of specificity of PCR primers.

Isolates	Number of the isolates^d^	Targeted gene loci of bacteria						
		ipaH	tlh	eaeA	invA	ail	ctxA	mapA
*Salmonella enteritidis *	CMCC^a^ 50041	−	−	−	+	−	−	−
*Shigella flexneri *	CMCC 51537	+	−	−	−	−	−	−
*Shigella sonnei *	2	+	−	−	−	−	−	−
EIEC	1	+	−	−	−	−	−	−
EHEC	3	−	−	+	−	−	−	−
EPEC	2	−	−	+	−	−	−	−
*Vibrio parahaemolyticus *	CICC^b^ 21617	−	+	−	−	−	−	−
*Vibrio cholera* O1	2	−	−	−	−	−	+	−
*Vibrio cholera* O139	1	−	−	−	−	−	+	−
*Yersinia enterocolitica *	3	−	−	−	−	+	−	−
*Campylobacter. jejuni *	4	−	−	−	−	−	−	+
*Vibrio mimicus *	CICC 21613	−	−	−	−	−	−	−
*Vibrio fluvialis *	3	−	−	−	−	−	−	−
*Staphylococcus aureus *	ATCC^c^ 29213	−	−	−	−	−	−	−
*Listeria monocytogenes *	CMCC 54004	−	−	−	−	−	−	−

^a^CMCC: National Center for Medical Culture Collection; ^b^CICC: China Center of Industrial Culture Collection; ^c^ATCC: American Type Culture Collection; ^d^the isolates in this panel consisted of clinical isolates and preserved standard strain.

**Table 3:** Primer sequences and product sizes used in this study.

Pathogen	Target	Sequence 5′-3′	Size	References
*Shigella* and EIEC	ipaH	AGGTGACACTATAGAATA ^ a^ACCATGCTCGCAGAGAAACT	213	[10]
		GTACGACTCACTATAGGGA ^ b^TCAGTACAGCATGCCATGGT		

*Vibrio parahaemolyticus *	tlh	AGGTGACACTATAGAATAACTCAACACAAGAAGAGATCGACAA	246	[30]
		GTACGACTCACTATAGGGAGATGAGCGGTTGATGTCCAA		

EHEC and EPEC	eaeA	AGGTGACACTATAGAATAAGGTCGTCGTGTCTGCTA	293	[31]
		GTACGACTCACTATAGGGACCGTGGTTGCTTGCGTTTG		

*Salmonella *	invA	AGGTGACACTATAGAATAGTGAAATTATCGCCACGTTCGGGCAA	323	[32]
		GTACGACTCACTATAGGGATCATCGCACCGTCAAAGGAACC		

*Yersinia *	ail	AGGTGACACTATAGAATATAATGTGTACGCTGCGAG	389	[5]
		GTACGACTCACTATAGGGAGACGTCTTACTTGCACTG		

*Vibrio cholerae *	ctx	AGGTGACACTATAGAATAACAGTAACTTAGATATTGCTCCAG	507	This study
		GTACGACTCACTATAGGGAACCATTCTTAAAAGTAATGATAGCCA		

*Campylobacter jejuni *	mapA	AGGTGACACTATAGAATACTATTTTATTTTTGAGTGCTTGTG	627	[33]
		GTACGACTCACTATAGGGAGCTTTATTTGCCATTTGTTTTATTA		

Noroviruses GI	RDRP	AGGTGACACTATAGAATACGCTGGATGCGCTTCCATGA	162	[21]
		GTACGACTCACTATAGGGAGCAAGAGGGTCAGAAGCATT		

Rotavirus	VP6	AGGTGACACTATAGAATAAAGTCTTCCACATGGAGGT	210	Modified [21]
		GTACGACTCACTATAGGGAARRTTICCAATTCCTCCAGT		

Norovirus GII	RDRP	AGGTGACACTATAGAATACAGACAAGAGCCAATGTTCA	279	Modified [21]
		GTACGACTCACTATAGGGATTTCTAATCCAGGGGTCAATT		

Human astrovirus	ORF1a	AGGTGACACTATAGAATACGTCATTATTTGTTGTCATA	326	[21]
		GTACGACTCACTATAGGGACATGTGCTGCTGTTACTATG		

Enteric adenovirus	Hexon	AGGTGACACTATAGAATATGTACAAGCCAGNTGTAGCTC	388	This study
		GTACGACTCACTATAGGGAAAGCAGTAATTTGGCANTTCGT		

Human bocavirus 1^c^	VP1	AGGTGACACTATAGAATAAAACCCATCACTCTCAATGCTT	412	This study
		GTACGACTCACTATAGGGACAGTATGTCTTCTTTCTGGACG		

Human bocavirus 2	VP1	AGGTGACACTATAGAATAAAATCCACCACTATCCATGCTC	412	This study
		GTACGACTCACTATAGGGACGGTGTGTCTTCTTTCTGGTCT		

^a^Universal primers-F: AGGTGACACTATAGAATA; ^b^universal primers-R: GTACGACTCACTATAGGGA; ^c^the primers for HBoV1 and HBoV2 are equally mixed, the amplicon sizes of both PCR products are exactly the same, and the primers are located at the same position in the corresponding viral genome; this method is able to identify the presence of HBoV1 and HBoV2 but cannot classify the subtypes.

**Table 4:** Detection of 13 enteric agents in 122 specimens.

Pathogen	Qiaxcel+^b^ reference^a^+	Qiaxcel+ reference−	Qiaxcel− reference+	Qiaxcel− reference−	Sensitivity	Specificity	Agreement	Kappa value
Norovirus GI	5	0	1	116	83.33%	100%	99.18%	0.9048
Rotavirus	30	5	0	87	100%	94.57%	95.9%	0.8954
Norovirus GII	40	0	5	77	88.98%	100%	95.9%	0.9099
Human astrovirus	13	0	0	109	100%	100%	100%	1
Enteric adenovirus	13	2	0	107	100%	98.17%	98.36%	0.9194
Human bocavirus	5	0	0	117	100%	100%	100%	1
*Shigella* ^ c^	4	0	1	117	80%	100%	99.18%	0.8847
*Vibrio parahaemolyticus *	0	0	0	122				
EHEC and EPEC	1	0	0	121	100%	100%	100%	1
*Salmonella *	3	0	0	119	100%	100%	100%	1
*Yersinia *	0	0	0	122				
*Vibrio cholerae *	0	0	0	122				
*Campylobacter jejuni *	0	0	0	122				

^a^The definition of “reference results” was described in “Virus, Strains, and Clinical Samples.” Virus was identified using a commercial ELISA kit, reported multiplex PCR assay, and monoplex PCR followed by sequencing at DD-IVDC [13–16]. All specimens were processed by routine isolation/culture to identify different enteropathogenic bacteria at DD-ICDC. The diarrheagenic *Escherichia coli* were identified using multiplex real-time PCR assay [17].

^b^The numbers of positive and negative specimens detected by the two-tube assay were indicated as Qiaxcel+ and Qiaxcel−, respectively. The numbers of positive and negative specimens detected by the reference assay were indicated as reference+ and reference−, respectively.

^c^The two-tube assay was not able to distinguish *Shigella* from EIEC, so *Shigella* positive detected by the two-tube assay could be *Shigella* or EIEC. These 5 samples (reference+) were confirmed by sequencing.

**Table 5:** Multiple enteric pathogens detected in clinical samples by novel two-tube assay.

Pathogen	Numbers of coinfection
Norovirus G2 and astrovirus	4
Adenovirus and astrovirus	3
Rotavirus and *Salmonella *	3
Rotavirus, astrovirus, and norovirus G2	3
Rotavirus, astrovirus, and adenovirus	2
Norovirus G2 and adenovirus	2
Rotavirus and astrovirus	1
Rotavirus and adenovirus	1
Rotavirus and *Shigella *	1
Rotavirus and norovirus G2	1

Total	21
